# Mediators linking gut microbiota and sporadic Creutzfeldt–Jakob disease: a Mendelian randomization study

**DOI:** 10.1186/s13568-025-01932-3

**Published:** 2025-08-18

**Authors:** Jie Shao, Tengfei Su, Jinyan Wang, Xiang Yin, Yue Lang, Yuxin Fu, Li Cui

**Affiliations:** 1 Department of Neurology, The First Hospital of Jilin University, Changchun, China; 2 Department of Pulmonary Disease, The Zhongwei Hospital of Traditional Chinese Medicine, Zhongwei, China

**Keywords:** Gut microbiome, Sporadic Creutzfeldt–Jakob disease, Mendelian randomization

## Abstract

While gut microbiome associations with sporadic Creutzfeldt–Jakob disease (sCJD) are recognized, causal mechanisms and mediation via cerebrospinal fluid (CSF) metabolites remain unestablished. Using bidirectional Mendelian randomization (MR) with mediation analysis and integrating genome-wide association study (GWAS) summary statistics from gut microbiota (​​composition in the FINRISK 2002 prospective cohort, n = 5,959​​), CSF metabolites (from the Wisconsin Alzheimer's Disease Research Center ​​Registry and Wisconsin Registry for Alzheimer's Prevention​​, n = 291), and sCJD case–control data (5,208 cases vs. 511,675 controls), we identified five microbial taxa influencing sCJD risk. Protective effects were observed for the family *Atopobiaceae* [odds ratio (OR) = 0.527, 95% confidence interval (CI) = 0.321–0.864, *P* = 0.011], the species *Enterococcus faecalis* (OR = 0.647, 95% CI = 0.427–0.980, *P* = 0.040), and the genus *Lactobacillus (group B)* (OR = 0.768, 95% CI = 0.602–0.981, *P* = 0.035). Conversely, the species *Bacteroides eggerthii* (OR = 1.228, 95% CI = 1.027–1.468, *P* = 0.025) and the order *Chloroflexales* (OR = 3.455, 95% CI = 1.214–9.835, *P* = 0.020) were pathogenic. Mediation analysis revealed that S-methylcysteine mediates 8.8% of ​​the effect of order *Chloroflexales* on sCJD risk​​, establishing it as a significant biological mediator in this pathogenic pathway. These findings provide novel biomarkers for early sCJD risk stratification, identify ​​the family *Atopobiaceae*, the species *Enterococcus faecalis*, and the genus *Lactobacillus (group B)*​​ as probiotic candidates for primary prevention, reveal S-methylcysteine pathway modulation as therapeutic entry points, and establish mechanistic foundations for disrupting gut-CSF transmission​​ in prion diseases.

**Graphical abstract:**

Bidirectional causal relationship between the gut microbiome and sporadic Creutzfeldt–Jakob disease (sCJD). Prefixes f., g., s., and o. in taxon labels denote family, genus, species, and order, respectively. The analysis encompassed 471 bacterial taxa across 11 phyla, 19 classes, 24 orders, 62 families, 146 genera, and 209 species. Abbreviations: WADRC, Wisconsin Alzheimer’s Disease Research Center; WRAP, Wisconsin Registry for Alzheimer’s Prevention; CSF, cerebrospinal fluid.
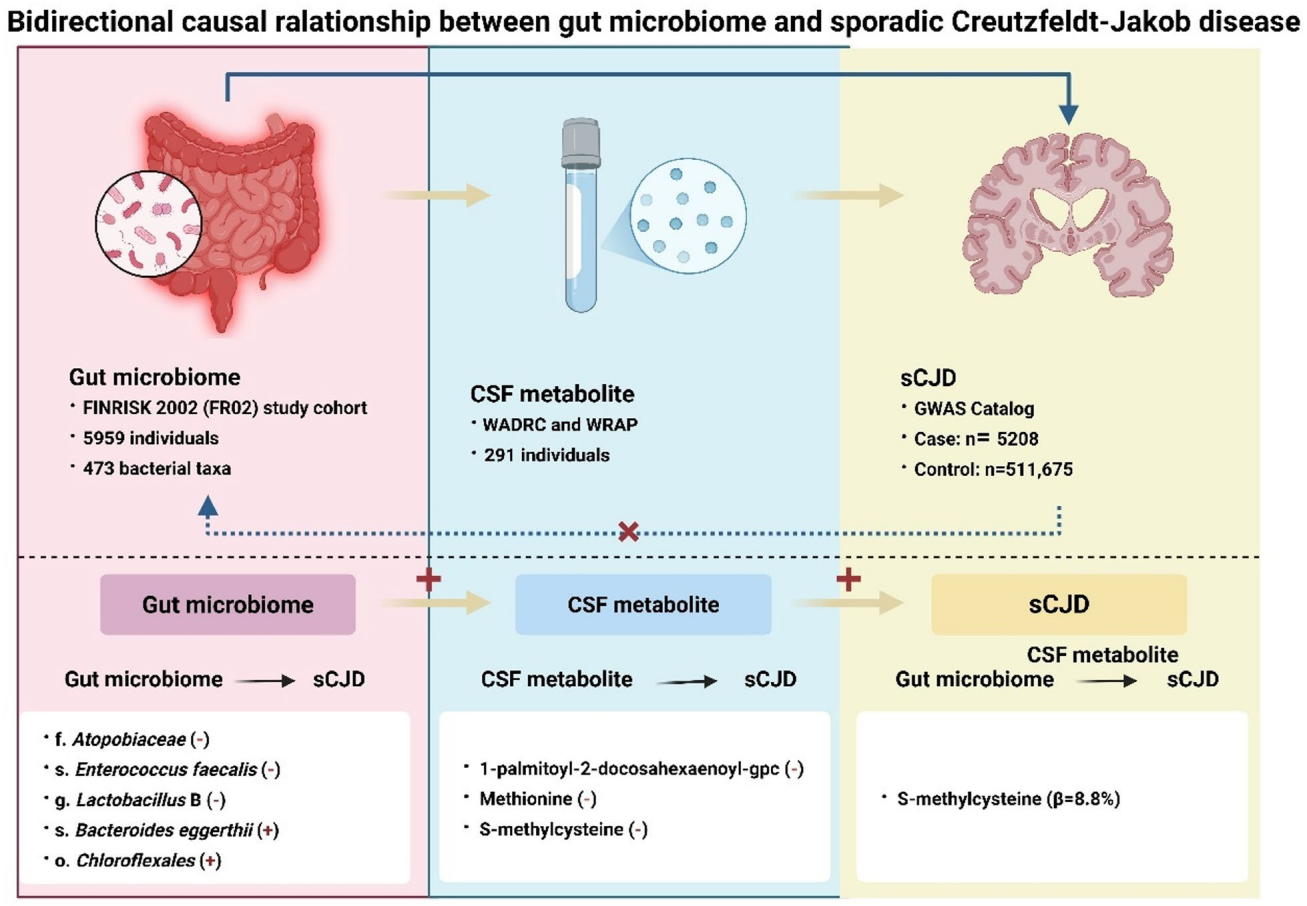

**Supplementary Information:**

The online version contains supplementary material available at 10.1186/s13568-025-01932-3.

## Introduction

Creutzfeldt–Jakob disease (CJD) is a rare, fatal, and transmissible neurodegenerative disorder caused by the deposition of misfolded prion protein (PrP^Sc^) (Ladogana et al. 2005). Sporadic CJD (sCJD) accounts for the majority (85%) of cases, yet its etiology remains poorly understood, and no effective treatments exist, leading to rapid progression and a median survival of under two years post-diagnosis (Hermann et al. 2021; Ladogana et al. 2005).

The gut microbiota is a key environmental modulator implicated in the pathogenesis of sCJD (Pineiro-Ramos et al. 2021). Supporting this link, germ-free mice exhibit prolonged survival following prion infection compared to conventional controls (Lev et al. 1971). Crucially, ​​distinct​​ gut microbiota dysbiosis patterns are observed in both murine models and sCJD patients: Yang et al. (2020a) reported increased *Proteobacteria* and *Erysipelotrichia* abundance in prion-infected mice, while Guo et al. (2022) identified enriched *Fusobacterium*, *Succinivibrio*, *Enterococcus*, *Ruminococcus gnavus*, and *Tyzzerella 4*, alongside depleted *Coprococcus 1*, *Lachnospiraceae_ND3007*, *Pseudobutyrivibrio*, *Roseburia*, and *Holdemanella* in sCJD patients. These alterations correlate with cognitive decline (Montreal Cognitive Assessment scores) and reduced survival duration (Guo et al. 2022; Kong et al. 2023). Furthermore, gut microbiota-mediated microglial inflammation contributes to amyloid formation in prion pathogenesis, reinforcing the critical role of gut microbiota in sCJD pathophysiology (D'Argenio and Sarnataro 2019). While our prior MR study robustly established a causal relationship between gut microbiota composition and sCJD risk (Su et al. 2025), the precise mechanisms underlying this gut-brain axis communication remain elusive. A critical gap exists in understanding the mediating pathways bridging gut dysbiosis to sCJD pathogenesis.

Emerging evidence strongly points to ​​cerebrospinal fluid (CSF) metabolites​​ as plausible mediators. Aberrant CSF metabolic profiles correlate tightly with sCJD progression. Notably, Kong et al. (2023) identified significant alterations in CSF metabolites, particularly reductions in phenylpropanoid biosynthesis pathway components like Biotin, in sCJD patients compared to healthy controls. These metabolites are potential products of gut microbial metabolism, positioning CSF metabolomic alterations as key candidates for mediating the observed causal effect of the gut microbiota on sCJD.

To directly address this critical knowledge gap—namely, the mediating role of CSF metabolites in the gut microbiota-sCJD causal axis—this study employs a rigorous methodological approach.​​ We leverage a ​​two-sample bidirectional MR framework coupled with mediation analysis.​​ This design integrates genetic instrumental variables (from GWAS identifying SNPs associated with gut microbiota, CSF metabolites, and sCJD) and CSF biomarker data to achieve two primary objectives: (1) Confirm and refine the causal relationship​​ between gut microbiota and sCJD; (2) Quantify the mediation effects​​ exerted by specific CSF metabolites along this causal pathway. By capitalizing on the inherent confounding resistance property of MR, this approach aims to elucidate the causal mechanisms linking gut dysbiosis to sCJD, specifically identifying and quantifying the role of CSF metabolites as mediators. This addresses a fundamental gap in understanding and identifies potential diagnostic/prognostic biomarkers or therapeutic targets.

## Method

### Study design

This two-sample bidirectional MR framework investigated causal relationships among gut microbiota, CSF metabolites, and sCJD under three methodological principles: (1) genetic variation is directly associated with the exposure; (2) genetic variants must be independent of confounders; (3) genetic variation does not influence the outcome through alternative pathways.

**Fig. 1:**
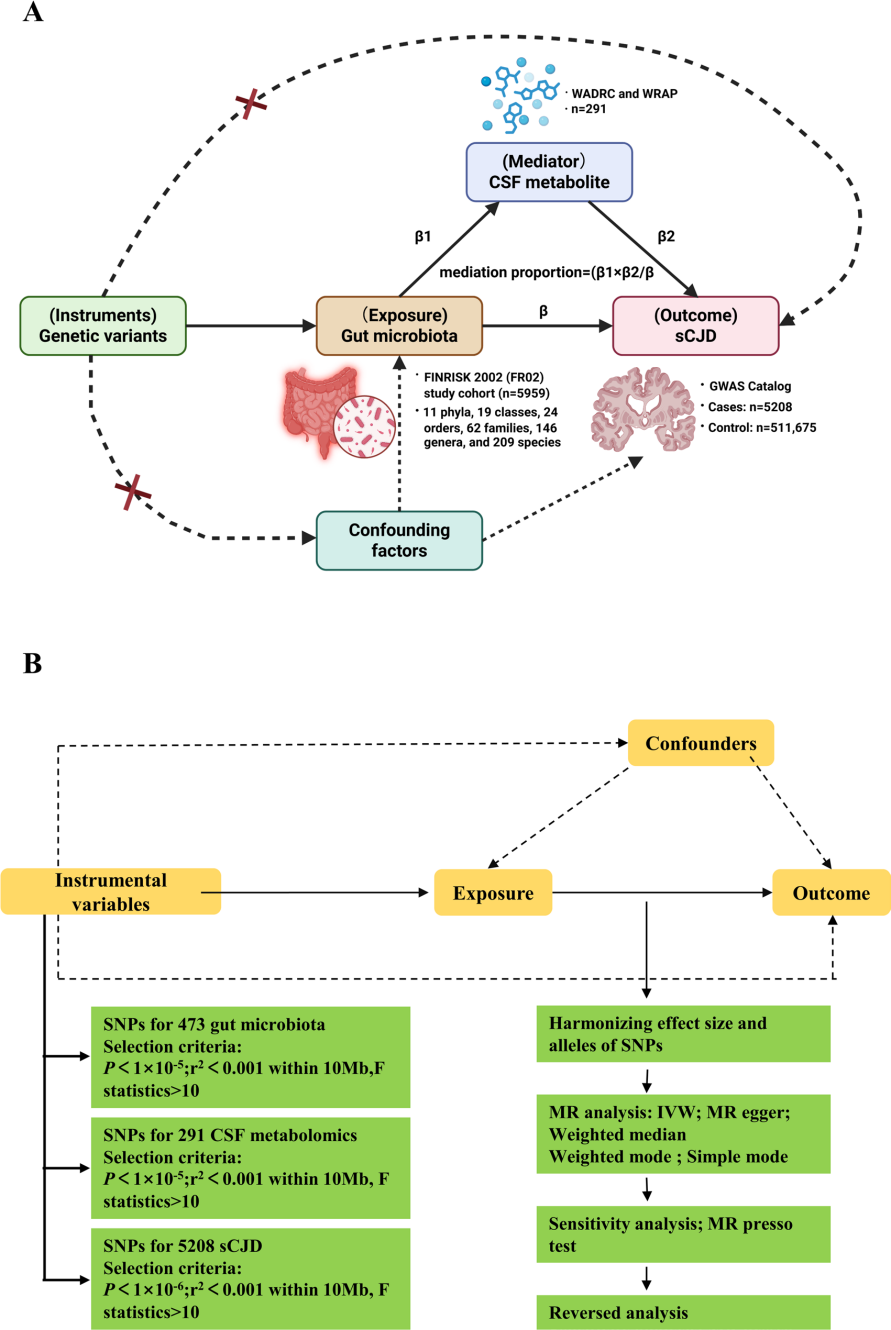
Conceptual framework and analytical workflow for bidirectional and mediation Mendelian randomization (MR) analyses. (A) Study design. First, bidirectional MR framework was implemented to evaluate causal relationships between gut microbiota (exposure) and sporadic Creutzfeldt-Jakob disease (sCJD; outcome). Subsequently, 291 cerebrospinal fluid (CSF) metabolite profiles were selected as potential mediators for pathway analysis. A two-step MR approach was then employed: Step 1 assessed gut microbiota's causal influence on CSF metabolites; Step 2 examined CSF metabolite-mediated effects on sCJD. Finally, multivariate MR analysis validated identified mediation pathways. (B) Data extraction workflow. 1) P-value Thresholds: Applied adjusted p-value thresholds: P < 1×10⁻⁵ (gut microbiota/CSF metabolites) and P<5×10⁻⁶ (sCJD). 2) Linkage disequilibrium (LD) Clumping: Performed LD-based clumping (window size= 10,000 kb, r² < 0.001) to retain independent SNPs. 3) Palindromic SNP Removal: Ensured allele direction consistency. 4) Instrumental variable Strength Validation: Quantified IV strength using F-statistics (F = β² /SE²), where β = effect allele coefficient, SE = standard error.

The analysis employed a two-stage design, as shown in Fig. 1A: Stage 1​​: Forward-directional MR treating ​​gut microbiota taxa or CSF metabolites as exposures​​ with sCJD as outcome, complemented by ​​reverse-directional MR​​ using sCJD-associated SNPs to test reverse causality, thereby identifying unidirectional associations. Stage 2​​: Mediation analysis quantifying microbiota-metabolite-sCJD pathways through the mediation proportion (β1 × β2 / β), ​​where​​: β1 represents the microbiota-metabolite effect, β2 denotes the metabolite-sCJD effect, ​​and​​ β indicates the total microbiota-sCJD association.

### Data sources and study participants

The summary statistics for gut microbiota data, CSF metabolites, and sCJD phenotype were obtained from previous GWAS studies, ​as detailed in Supplementary Table S1​​.

GWAS data on gut microbiome composition were generated through shotgun metagenomic sequencing of stool samples from 5,959 European-descent participants in the Finnish FINRISK 2002 cohort. The analysis encompassed 2,801 microbial taxa spanning 11 phyla, 19 classes, 24 orders, 62 families, 146 genera, and 209 species. Detailed taxonomic information, including taxonomic identifiers​​​​ and full ​​Genome Taxonomy Database (GTDB)​​ classification pathways, is provided in ​​Supplementary Tables S9-S10.

GWAS data on CSF metabolomic profiles​​ were derived from secondary analysis of biospecimens from the Wisconsin Alzheimer’s Disease Research Center and Wisconsin Registry for Alzheimer’s Prevention cohorts (n = 291, European ancestry).

The GWAS data on sCJD were obtained from patients diagnosed with probable or definite sCJD, collated through specialist clinics and national surveillance centers in predominantly European-ancestry populations.

### Genetic instrument optimization​​

This study implemented a three-stage quality control pipeline for SNP selection, as depicted in Fig. 1B. (1) P-value thresholds for trait associations: Applied *P* < 1 × 10⁻^5^ for microbiota/metabolite traits and *P* < 5 × 10^−6^ for sCJD. (2) LD clumping​​: Performed LD clumping (window size = 10,000 kb; *r*^*2*^ < 0.001) to select independent SNPs, followed by removal of palindromic SNPs to prevent allele strand ambiguity. (3) ​​Instrument strength validation​​: Calculated F-statistics (*F* = β^2^/ SE^2^), where β = effect allele coefficient and SE = standard error.

### Statistical analysis

The primary MR analysis was conducted using the inverse-variance weighted (IVW) method, complemented by MR-Egger, weighted median, weighted mode, and simple mode to ensure robustness (Bowden et al. 2015; Hemani et al. 2018). Heterogeneity assessment employed Cochran's Q statistic, while horizontal pleiotropy was addressed through MR-Egger regression. Outlier detection and removal utilized MR-PRESSO and leave-one-out analyses to mitigate bias. Visual outputs included forest plots, circular genome maps, and phylogenetic trees for data interpretation. SNPs with F-statistics ≤ 10 (indicating weak instrumental variables) were excluded a *priori*. Multiple testing correction applied Bonferroni's method, with adjusted *P* < 0.05 indicating suggestive associations.

CSF metabolite mediators were selected based on three criteria: (1) unidirectional exposure-mediator relationship; (2) concordance between metabolite-mediated direction and overall exposure-outcome association; (3) statistically significant exposure-mediator and mediator-outcome effects. This two-step MR framework quantified the mediating role of CSF metabolites in gut microbiota-sCJD associations. The mediation proportion was calculated as the ​​indirect-to-total effect ratio (β₁ × β₂ / β)​​, where: β_1_ = microbiota-CSF metabolite association; β_2_ = CSF metabolite-sCJD effect; β = total microbiota-sCJD effect. Bootstrap-derived standard errors​​ and ​​two-sample MR effect estimates​​ provided statistical robustness. All analyses were executed in ​​R (version 4.4.2)​​ using the ​​TwoSampleMR package (v0.6.8)​​.

### Ethical approval and consent

This study utilized publicly available data. Each constituent study within the GWAS meta-analyses had obtained institutional review board approval and written informed consent from participants or their caregivers, legal guardians or authorized representatives.

## Result

### Instrumental variables included in the analysis

Instrumental variables encompassed SNPs associated with​​ gut microbiota composition, CSF metabolite profiles, and sCJD risk, with comprehensive characteristics detailed in online supplementary Tables S2-S3. These IVs demonstrated robust instrument strength​​ (F-statistic > 10), minimizing​​ weak instrument bias while ensuring reliable causal inference.

### Causal associations of gut microbial taxa with sCJD

**Fig. 2:**
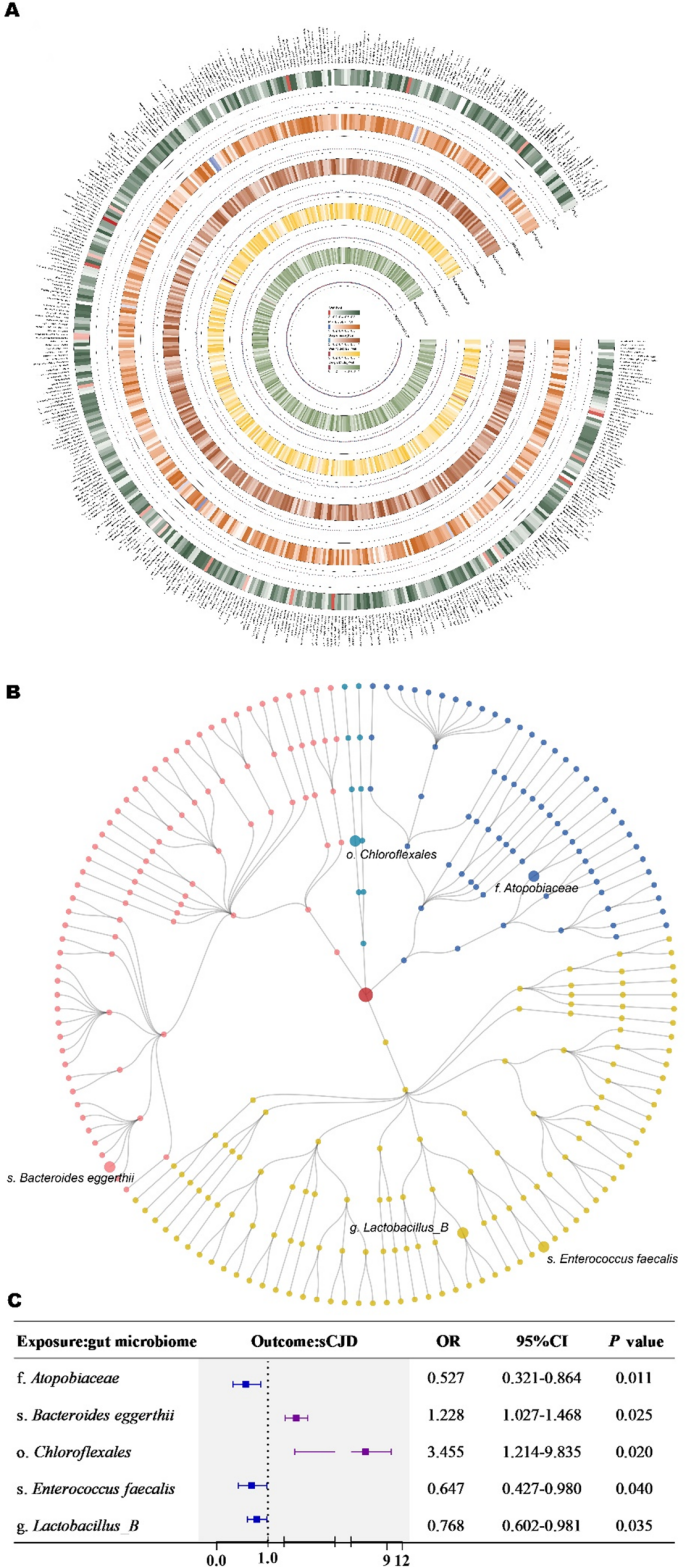
Mendelian randomization analysis of the causal effects of gut microbiota on sCJD. **A** Circos diagram illustrating statistically significant causal effects of gut microbiota taxa on sCJD risk. Concentric rings display odds ratios (ORs) and *P*-values from five Mendelian randomization methods (IVW, MR-Egger, simple mode, weighted median, weighted mode), color gradients represent​​​​ *P*-value significance. **B** Taxonomic classification​​ of microbial taxa included in the current study (*P* < 0.05). Each dot represents a microbial taxon. The main branches represent different microbial phyla, each phylum is shown in a distinct color​​​​. Within each phylum, branches radiating outward represent hierarchical classifications (class – order—family—genus—species). Taxa showing suggestive significance in this study are labeled. **C** Forest plot​​ of causal estimates between microbial taxa and sCJD. Odds ratios and 95% confidence intervals were derived using the inverse-variance weighted method. The prefixes f., g., s., and o. in the taxon column denote ​​family, genus, species, and order​​, respectively. Abbreviations: OR, odds ratio; CI, confidence interval; IVW, inverse-variance weighted

We identified five microbial taxa causally associated with sCJD based on the IVW method, as depicted in Fig. 2. Specifically, increased abundance of​​ family *Atopobiaceae* [odds ratio (OR) = 0.527, 95% confidence interval (CI) = 0.321–0.864,* P* = 0.011], species *Enterococcus faecalis* (OR = 0.647, 95% CI = 0.427–0.980, *P* = 0.040), and genus *Lactobacillus * B (OR = 0.768, 95% CI = 0.602–0.981, *P* = 0.035) were ​associated with reduced​​ sCJD risk. Conversely, elevated abundance of species *Bacteroides eggerthii* (OR = 1.228, 95% CI = 1.027–1.468, *P* = 0.025) and order *Chloroflexales* (OR = 3.455, 95% CI = 1.214–9.835, *P* = 0.020) showed ​​positive associations with sCJD risk. These findings were consistent across four MR methods, as detailed in supplementary Tables S4 and S7.

Reverse-direction IVW analysis assessed causal effects of sCJD on gut microbiota, revealing no significant associations: family *Atopobiaceae* (OR = 0.994, 95% CI = 0.967–1.022, *P* = 0.673), species *Bacteroides eggerthii* (OR = 0.973, 95% CI = 0.887–1.068, *P* = 0.568), order *Chloroflexales* (OR = 0.993, 95% CI: 0.969–1.019, *P* = 0.600), species *Enterococcus faecalis* (OR = 0.969, 95% CI: 0.780–1.024, *P* = 0.647), and genus *Lactobacillus B* (OR = 1.045, 95% CI: 0.996–1.095, *P* = 0.072). These findings were corroborated by four additional MR methods, as detailed in supplementary Tables S5.

Collectively, these results suggest a unidirectional causal relationship from gut microbiota to sCJD.

### Causal associations of CSF metabolites with sCJD

Our analysis identified three CSF metabolites exhibiting causal associations with sCJD risk (Table 1 and Supplementary Table S8). Specifically, elevated levels demonstrated protective effects: 1-palmitoyl-2-docosahexaenoyl-gpc (16:0/22:6) (OR = 0.898, 95% CI: 0.827–0.975, *P* = 0.011), methionine (OR = 0.763, 95% CI = 0.599–0.973, *P* = 0.029), and S-methylcysteine (OR = 0.931, 95% CI = 0.871–0.994, *P* = 0.032). Reverse MR analysis confirmed unidirectional causality from these metabolites to sCJD (Supplementary Table S6).

**Table 1:** Mendelian randomization analysis on the causal effect of the cerebrospinal fluid metabolites on sporadic Creutzfeldt–Jakob disease

Exposure	Method	No. SNP	Beta	*P*-value	OR	95% CI	Q statistic	Egger intercept	MR-PRESSO
1-palmitoyl-2-docosahexaenoyl-gpc	IVW	80	− 0.107	0.011	0.898	0.827–0.975	0.282	0.911	0.28
	MR-Egger	80	− 0.115	0.139	0.892	0.767–1.036	0.256	–	
	Weighted median	80	− 0.103	0.069	0.902	0.807–1.008	–		
	Simple mode	80	− 0.147	0.316	0.863	0.649–1.148	–		
	Weighted mode	80	− 0.152	0.287	0.859	0.651–1.134	–		
Methionine	IVW	44	− 0.270	0.029	0.763	0.599–0.973	0.678	0.105	0.712
	MR-Egger	44	− 0.559	0.012	0.572	0.376–0.870	0.751	–	
	Weighted median	44	− 0.346	0.045	0.707	0.504–0.993	–		
	Simple mode	44	− 0.455	0.191	0.634	0.324–1.242	–		
	Weighted mode	44	− 0.443	0.187	0.642	0.336–1.226	–		
S-methylcysteine	IVW	17	− 0.072	0.032	0.931	0.871–0.994	0.563	0.548	0.558
	MR-Egger	17	− 0.147	0.265	0.863	0.672–1.108	0.518	–	
	Weighted median	17	− 0.043	0.384	0.958	0.871–1.055	–		
	Simple mode	17	− 0.012	0.887	0.988	0.834–1.169	–		
	Weighted mode	17	− 0.016	0.867	0.984	0.822–1.179	–		

Odds ratio (OR), 95% confidence interval (CI), and *P*-values were calculated for the respective method of MR analysis. The heterogeneity test in the IVW methods was performed using Cochran’s *Q* statistic and the global test for the MR-PRESSO method

IVW, inverse–variance weighted; *P*-heterogeneity, *P*-value for heterogeneity test; *P*-intercept, *P*-value for the intercept of MR-Egger regression; *P*-value for MR-PRESSO

### Metabolites in causal species between gut microbiota and sCJD

**Fig. 3:**
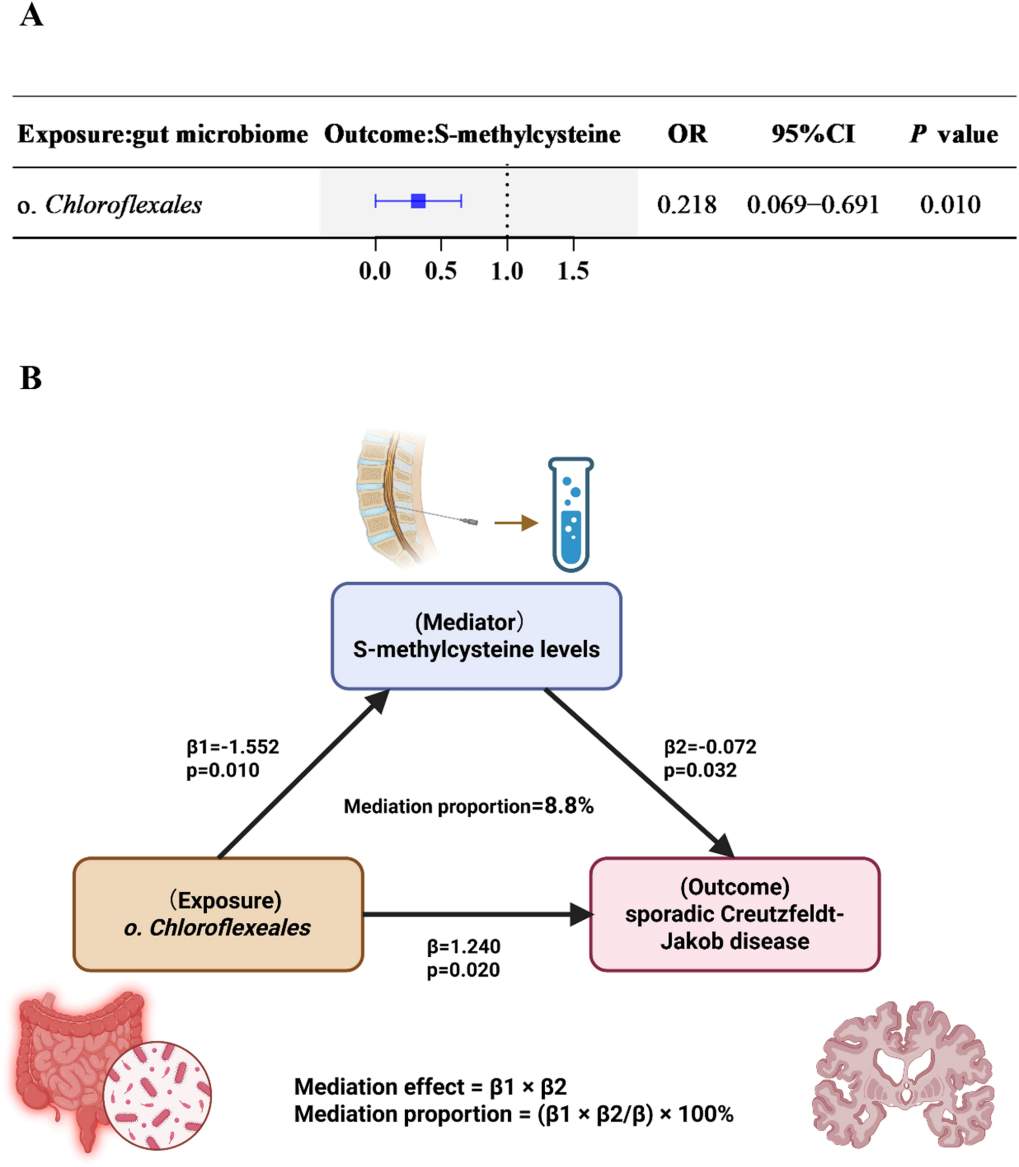
S-methylcysteine mediated the causal effect of order *Chloroflexales* on sCJD. **A** Forest plot of causal estimates between microbial taxa and CSF metabolites. The odds ratio (OR) and 95% confidence interval (CI) were derived using the inverse-variance weighted (IVW) method. Abbreviations: OR, odds ratio; CI, confidence interval. **B** The β value between order *Chloroflexales* and S-methylcysteine and sCJD is the MR estimates using the IVW method. The β value between S-methylcysteine and sCJD is the Mendelian randomization estimates using the Mendelian randomization pleiotropy residual sum and outlier method.

We first applied the IVW method to analyze associations between five gut microbiota taxa and three CSF metabolites, as depicted in Fig. 3A. The results revealed a significant negative correlation between order *Chloroflexales* and S-methylcysteine (OR = 0.218, 95% CI = 0.069–0.691, *P* = 0.010). As a pathogenic taxon linked to sCJD, order *Chloroflexales* may exert detrimental effects by reducing S-methylcysteine levels, thereby exacerbating disease progression.

We subsequently performed multivariable MR to validate the mediating role of CSF metabolite S-methylcysteine identified via two-sample MR, as depicted in Fig. 3B. The analysis revealed a significant indirect effect of order *Chloroflexales* on sCJD risk mediated through S-methylcysteine, with a mediation proportion of 8.8%.

## Discussion

This large-scale and comprehensive MR study identified five gut microbiota taxa demonstrating causal associations with sCJD, including order *Chloroflexales*. Mediation analysis revealed that S-methylcysteine may mediate 8.8% of the causal effect of order *Chloroflexales* on sCJD. Our MR analysis of the microbiota-metabolite-disease axis provided evidence for gut microbiota's causal role in sCJD and confirmed CSF metabolites' mediating function.

The cellular prion protein (PrP^C^), a membrane glycoprotein abundant in neurons, undergoes pathogenic misfolding into the β-sheet-rich isoform PrP^Sc^ in prion diseases (Miranzadeh Mahabadi and Taghibiglou 2020; Cha and Kim 2023; Baral et al. 2019). This core event initiates a self-propagating cascade where PrP^Sc^ templates further misfolding of native PrP^C^ (Hackl and Becker 2019), ultimately forming neurotoxic amyloid aggregates that drive neurodegeneration (Willbold et al. 2021).

Emerging evidence highlights a potential link between gut microbiota dysbiosis and prion disease pathogenesis. Patients with sCJD exhibit altered gut microbiota profiles characterized by increased *Fusobacterium*, *Enterococcus*, *Ruminococcus*, and *Tyzzerella* genera abundance alongside reduced *Pseudobutyrivibrio*, *Roseburia*, and *Holdemanella* abundance compared to healthy controls (Guo et al. 2022). Further mechanistic studies propose dual pathways: (1) PrP^Sc^ interacts with bacterial curli proteins, which act as amyloid fibril templates through cross-seeding events, accelerating protein misfolding and propagation (Kushwaha et al. 2023; Sleutel et al. 2023); (2) Dysbiosis compromises intestinal barrier integrity, facilitating prion migration from gut to brain (Donaldson et al. 2012; Kushwaha et al. 2023; Shu et al. 2023). Our MR analyses identified five gut microbiota taxa causally linked to sCJD—protective Gram-positive family *Atopobiaceae*, species *Enterococcus faecalis*, and genus *Lactobacillus* B inversely correlate with sCJD risk, while pathogenic Gram-negative species *Bacteroides eggerthii* and order *Chloroflexales* show positive associations. Yoshioka demonstrated that proteases secreted by Gram-positive bacteria degrade PrP^Sc^ in scrapie and bovine spongiform encephalopathy more efficiently than proteinase K and PWD-1 (Yoshioka et al. 2007). Moreover, another distinguishing feature of prion diseases is the neuroinflammation caused by alterations in the gut microbiota. For instance, overgrowth of Gram-negative bacteria leads to massive secretion of endotoxins, which are lipopolysaccharides and classical pro-inflammatory mediators. Research demonstrates that lipopolysaccharides potently activate microglia via the toll-like receptor 4 signaling pathway, driving the secretion of nitric oxide, reactive oxygen species, and cytokines that damage neurons (Skrzypczak-Wiercioch and Salat 2022; Yang et al. 2020a, b). Furthermore, endotoxin binding to apolipoprotein E promotes amyloid-β and tau aggregation, increasing Alzheimer’s disease susceptibility (Brown and Heneka 2024). In a mouse model of Parkinson's disease, endotoxin induces α-synuclein production/aggregation and leads to loss of dopaminergic neurons in the substantia nigra (Gao et al. 2011). Elevated blood endotoxin levels in amyotrophic lateral sclerosis patients correlate with accelerated TAR-DNA binding protein of 43 kDa aggregation and neuropathology (Correia et al. 2015). While the endotoxin hypothesis in sCJD remains unproven, therapeutic strategies targeting endotoxin levels or endotoxin-induced neuroinflammation could theoretically mitigate disease sCJD progression. In addition, Jereme observed a strong pro-oxidant environment and altered redox metal homeostasis in the early disease pathology of prion disease, both of which may be contributing factors that exacerbate this protein misfolding disease (Spiers et al. 2022).

The gut microbiota, often termed the “second endocrine organ,” modulates host metabolism, inflammation, and other physiological/pathological processes through the production of metabolites and bioactive small molecules (Erny et al. 2021). Our large-scale MR analysis identified S-methylcysteine as an important mediator in the causal relationship between gut microbiota and sCJD risk. S-methylcysteine, an organosulfur compound abundant in garlic, exhibits potent antioxidant and anti-inflammatory properties (Elmahallawy et al. 2021). Exogenous supplementation of antioxidants significantly ameliorates oxidative stress and mitochondrial dysfunction in PrP106-126-treated N2a neuronal cells (Wen et al. 2025). Furthermore, a two-sample bidirectional MR study linking CSF metabolites to Parkinson’s disease revealed an inverse association between CSF S-methylcysteine and Parkinson’s disease risk (Liao et al. 2025). Dietary supplementation with S-methylcysteine has also been shown to prevent α-synuclein-induced pathogenic aggregates (Wassef et al. 2007). Collectively, these findings propose antioxidant therapy as a novel and promising therapeutic strategy for prion diseases. Of course, the partial mediating role of S-methylcysteine (8.8%) indicates the involvement of potential complementary pathways influenced by unmeasured factors, such as other metabolites (e.g., short-chain fatty acids) or inflammatory cytokines. These future strategies are warranted: (1) multi-omics mechanistic exploration​​ through combined metabolomic-proteomic profiling to elucidate complementary pathways involving unmeasured factors (e.g., short-chain fatty acids, inflammatory cytokines); (2) ​​Prospective cohort studies establishing temporal associations, modulation of S-methylcysteine in human prion transmission models to quantify anti-aggregatory effects, Probiotic interventions targeting causal microbial taxa (e.g., Chloroflexales).

Mediation MR studies offer a practical and robust framework for dissecting the contribution of risk factors to disease pathogenesis. By identifying mediators along causal pathways from risk factors to disease endpoints, these methods enable precise therapeutic target identification and facilitate the development of interventions to mitigate adverse exposure outcomes. In the context of gut microbiota-driven sCJD, mediation analysis revealed S-methylcysteine as a CSF metabolite-candidate biomarker. These findings advance our understanding of microbiota-sCJD molecular mechanisms while informing the development of microbiota-modulating therapeutics. This paradigm shift positions gut microbiota-targeted strategies as a promising frontier for innovative sCJD treatment paradigms.

This study offers several advantages. Unlike observational studies, the MR design establishes causal relationships between exposures and outcomes rather than mere associations. However, our study has limitations: (1) The unavailability of publicly accessible individual-level GWAS data precluded evaluation of non-linear dose-response relationships between gut microbiota/CSF metabolites and sCJD risk, while simultaneously limiting covariate adjustment for individual-level confounders (e.g., diet, comorbidities). Future studies should incorporate individual-level data from prospective cohorts with detailed covariate ascertainment to address these constraints; (2) the inclusion of GWAS data solely from individuals of European ancestry limits generalizability to other populations, and future validation in multi-ethnic cohorts is required; (3) MR analysis may incompletely capture the time-varying nature of gut microbiota and CSF metabolites, recognized as a current methodological challenge in MR studies (Labrecque and Swanson 2020); (4) the absence of suitable prospective observational data restricts replication through individual-level analyses; ​(5) The low incidence of sCJD substantially limits robust subgroup analyses. Future multicenter collaborative initiatives are needed to integrate data and assemble sufficiently large datasets, thereby enabling exploration of potential heterogeneity in gut microbiota-sCJD associations; (6) MR assumes relative stability of genetic effects over time, potentially missing dynamic associations influenced by age, disease progression, or environmental exposures. This 'time-invariant' assumption may not fully capture longitudinal variations in microbial/metabolite profiles.​ Addressing these limitations necessitates future methodological advancements for effective temporal modeling and longitudinal cohort studies with extended follow-up to overcome these constraints.

In conclusion, this study identifies a single CSF metabolite as a mediator of gut microbiota-driven sCJD. These findings advance our understanding of the mechanistic interplay between gut microbiota and sCJD pathogenesis. Notably, S-methylcysteine emerges as a potential biomarker and therapeutic target for microbiota-driven sCJD, offering novel insights for disease prevention and targeted intervention strategies.

## Conclusion

To our knowledge, this represents the first comprehensive assessment of causal relationships among gut microbiota, CSF metabolites, and sCJD. Our findings demonstrate a causal link between specific gut microbial taxa (e.g., order *Chloroflexales*) and sCJD risk, while identifying S-methylcysteine as a key CSF metabolite mediator. These results not only underscore the critical importance of underlying mechanisms driving microbiota-sCJD interactions but also provide novel insights for developing microbiome-targeted therapies and metabolite-based therapeutic strategies to mitigate prion disease progression.

## Supplementary Information

Below is the link to the electronic supplementary material.

Supplementary Material 1

## Data Availability

Data is provided within the supplementary information files.

## Abbreviations

sCJD: Sporadic Creutzfeldt–Jakob disease
MR: Mendelian randomization
CSF: Cerebrospinal fluid
GWAS: Genome-wide association study
IVW: Inverse-variance weighted
OR: Odds ratio
CI: Confidence interval
PrP^Sc^: Misfolded prion protein
SNPs: Single nucleotide polymorphisms
LD: Linkage disequilibrium
β: Beta
SE: Standard error
PrP^C^: Cellular prion protein
